# Supplementary material for: A review of HPV and HBV vaccine hesitancy, intention, and uptake in the era of social media and COVID-19
Source: eLife. 2023 Aug 18;12:e85743. doi: 10.7554/eLife.85743 (PMC10438906; doi:10.7554/eLife.85743)
Supplement: Supplementary file 1. — (a) Original search. Conducted on October 22, 2022. Includes articles made available in the databases through October 22, 2022. (b) Bridge review. Conducted on June 6, 2023. Includes articles made available in the databases from October 22, 2022, to May 31, 2023. [file elife-85743-supp1.docx]

**Supplemental Appendix:** Results of combined search, original search, and bridge search

**Supplementary file 1a: Original search.** Conducted on Oct. 22, 2022. Includes articles made available in the databases through Oct. 22, 2022**.**

| **Step** | **Search Terms** | **Medline** | **PsychInfo** |
| --- | --- | --- | --- |
| 1 | (misinformation or disinformation or conspiracy theory or rumor or fake news) | 6,248 | 12,908 |
| 2 | (social media) or (social network and online) or (social network and digital) or (social network and internet) | 31,155 | 63,944 |
| 3 | (COVID) or (SARS-CoV-2) | 304,144 | 32,614 |
| 4 | vaccine and (hesitancy or uptake or intention) | 14,105 | 4,856 |
| 5 | HPV or HBV | 97,923 | 6,459 |
| 6 | Research Question 1: Searches 3, 4, and 5 combined with the Boolean term “and” | 55 | 202 |
| 7 | Research Question 2: Searches 1, 2, 3, 4, and 5 combined with the Boolean term “and” | 5 | 49 |

**Supplementary file 1b: Bridge review.** Conducted on June 6, 2023. Includes articles made available in the databases from Oct. 22, 2022, to May 31, 2023.

| **Step** | **Search Terms** | **Medline** | **PsychInfo** |
| --- | --- | --- | --- |
| 1 | (misinformation or disinformation or conspiracy theory or rumor or fake news) | 920 | 1,703 |
| 2 | (social media) or (social network and online) or (social network and digital) or (social network and internet) | 4,216 | 8,614 |
| 3 | (COVID) or (SARS-CoV-2) | 55,844 | 16,382 |
| 4 | vaccine and (hesitancy or uptake or intention) | 2,206 | 805 |
| 5 | HPV or HBV | 3,425 | 414 |
| 6 | Research Question 1: Searches 3, 4, and 5 combined with the Boolean term “and” | 29 | 112 |
| 7 | Research Question 2: Searches 1, 2, 3, 4, and 5 combined with the Boolean term “and” | 2 | 39 |
